# Usability and Effects of a Combined Physical and Cognitive Intervention Based on Active Video Games for Preschool Children

**DOI:** 10.3390/ijerph19127420

**Published:** 2022-06-16

**Authors:** Ze-Min Liu, Chuang-Qi Chen, Xian-Li Fan, Chen-Chen Lin, Xin-Dong Ye

**Affiliations:** 1 Department of Educational Technology, Wenzhou University, Wenzhou 325035, China; iszemin.liu@gmail.com (Z.-M.L.); rico.chen9521@gmail.com (C.-Q.C.); 2 Department of Preschool Education, Wenzhou University, Wenzhou 325035, China; xianlicyc@gmail.com

**Keywords:** exergames, active video games, executive function, cognitive functions, physical activity, preschool children

## Abstract

Executive functions (EFs) are essential for early childhood development, and effective programs to improve EFs in preschool education are becoming increasingly crucial. There is rising evidence that combined physical–cognitive intervention training utilizing active video games (exergames) could be a viable strategy to improve EFs. However, there is a shortage of empirical evidence on the application of this approach in preschool education. The effectiveness of exergame intervention training in preschools must be evaluated. This study conducted a randomized controlled trial to assess the effects of exergames intervention training on preschool children’s EFs. A total of 48 participants aged 4–5 years were enrolled; 24 were randomly allocated to receive exergames physical activity training, and the remaining 24 received conventional physical activity training. After a four-week intervention, the children who received the exergames intervention training exhibited considerably greater gains in all three EFs tasks than children who received the conventional physical activity program. Follow-up interviews revealed that the children accepted the exergames well. The results demonstrate the viability of incorporating exergames into preschool education to improve children’s EFs, supporting prior findings and offering more empirical evidence from early childhood research.

## 1. Introduction

Executive functions (EFs) are a set of top-down cognitive processes that include three core EFs [1,2]: inhibitory control (inhibition), cognitive flexibility (shifting), and working memory (WM). The competencies possessed by EFs are crucial for intentional, goal-directed behavior and play an important role in cognitive and emotional self-regulation [2,3]. Due to the overlap between EFs and early school-age educational requirements (e.g., memory and extraction of information, inhibition of impulses to focus on learning, and multiple perspectives and flexible transitions), EFs are a better predictor of academic performance than intelligence for preschool children [2,4,5]. EFs are also closely connected with children’s cognitive development and mental health; for instance, several mental developmental disorders (e.g., Autism Spectrum Disorder and Attention Deficit Hyperactivity Disorder) are characterized by defective or deficient EFs [1,3]. In addition, EFs are highly connected with physical health, with poorer EFs being associated with obesity, binge eating, etc. [1], while an increase in EFs promotes healthy behaviors, with the relationship between the two being bidirectional [6]. Given the vital function of EFs in early childhood development and their importance for socio-emotional and cognitive development, the prospect of boosting these cognitive processes has garnered substantial interest [2,3].

To improve children’s executive functioning, a variety of interventions have been proposed [7], including computerized training, classroom sessions, and physical activities [8,9]. Children are in a critical period of rapid cognitive, social-emotional, and physical development [10]; factors such as excessive stress, loneliness, and lack of exercise can affect EFs development and can even cause damage to the cerebral cortex. Therefore, interventions that consider both their socio-emotional development (e.g., reducing classroom stress, stimulating interests, promoting social connections, etc.) and physical development (e.g., aerobics, exercise, yoga) are more beneficial [7]. Given their ecological validity, physical activities have been recommended as a promising approach to improving children’s EFs [11]. Diamond and Ling [12] further suggested that interventions requiring some cognitive involvement in physical activities are more effective than simple and repetitive activities that require less cognitive involvement. Schmidt et al. [13] came to the same conclusion in an intervention study with school-aged children aged 10 to 12 years. In intervention research with children aged 7 to 9, Egger et al. [14] discovered that a combination of high physical exertion and high cognitive engagement resulted in better shifting performance. This phenomenon could be explained by the presence of overlapping brain regions important for both abilities, such as the frontal, parietal, and motor cortices, which participate in both EFs and motor tasks, so that physical activities that combine cognitive involvement activate the same brain regions used to control EFs [8,11,15]. From a behavioral perspective, more cognitively engaged physical activities to necessitate stronger motor and cognitive control, and there is a clear overlap between the two notions, with the high demands of motor tasks necessitating the participation of EFs [16,17]. To summarize, the current evidence reveals the plasticity of EFs, and combining physical exercise with cognitively challenging tasks appears to be a viable strategy. However, lack of physical activity is currently widespread among children, with reports [18] indicating that nearly half of preschool children do not meet the recommended physical activity levels established by the American Academy of Pediatrics and that children face barriers to physical activity such as a lack of motivation, time constraints, and inadequate social support [19].

Video games have the potential to enhance children’s EFs. Video games are fun for children, and video games’ mechanics and feedback motivate users to repeat skills at increasing levels of difficulty and to maintain a high level of focus on the screen at all times [20]. Several studies have discovered that video games may have a positive impact on EFs [21,22]. However, excessive video game play can result in several negative side effects, such as increasing extended habitual sedentary behavior in children, displacing beneficial activities such as physical exercise [23], and causing visual [24] and other health issues. Simply restricting children’s video game play is frequently met with resistance. Another strategy is to try to utilize the same motivation to promote children’s interest in physical activity [19].

In recent years, researchers have become intrigued by the concept of active video games (exergames) which combine physical exercise with cognitively challenging tasks in an interactive game-based format [15,25,26]. Exergames, as opposed to traditional training programs, provide users with greater enjoyment and motivation through gamification mechanics [27], while also providing the adequate intensity of physical activity, such as ‘Just Dance’, which is considered to have a medium level of physical activity intensity [28]. Exergames are projected to provide ecologically beneficial combined physical–cognitive intervention training from an ecological standpoint [29,30]. However, empirical research in this field has been limited, with most studies focusing on interventions for children with developmental disorders [29], while studies assessing the impact of exergames on cognitive functioning in preschool children remain scarce [15,25,31]. The preschool years of 3-6 years are a critical period for the rapid development of individual EFs and the peak of individual brain development [10,32,33]. Therefore, focusing on the development of EFs and conducting programs to effectively increase EFs in preschool is an increasingly important area that can have a considerable impact on the success and future development of preschool children after school [2,34]. As a result, more research is necessary to determine the viability of combined physical–cognitive intervention training using exergames in preschool education.

To this end, a randomized controlled experiment was performed to evaluate the effects on preschool children’s EFs of combined physical–cognitive intervention training using exergames. After the intervention, researchers interviewed the preschool children who had received exergames training to assess their acceptance of exergames. The hypothesis to be tested is that preschool children in the exergames group would show a greater increase in EFs compared to those in the control group who received conventional physical activity training. In addition, preschool children in the exergames group were hypothesized to show a good acceptance of exergames.

## 2. Materials and Methods

### 2.1. Participants

Preschool children were recruited from a kindergarten in China. The study protocol was established with the approval of the school principal and teachers, and a list of normally healthy and developing children aged 4–5 years was obtained (i.e., normal development, no history of medical conditions that prevented exercise, and no diagnosed medical or psychological disorders that could affect the study results). The researchers personally contacted the children’s parents or guardians to present and explain the study protocol and also reconfirm the children’s health and developmental status. They also inquired about the children’s physical exercise/activity status outside of school (children who regularly engaged in physical exercise/activity of more than moderate intensity outside of school on one or more days per week were excluded at this stage). They excluded children who had played the “Just Dance” series of games. Finally, parents or guardians who consented to allow their children to participate signed informed consent forms.

A total of 48 preschool children (25 girls; Mean age = 4.90 ± 0.31 years) obtained written informed consent from their parents or guardians and completed the baseline EFs test. Of these individuals, 24 (12 girls; Mean age = 4.92 ± 0.28 years) were randomly allocated to the exergames group to receive the combined physical–cognitive intervention training via exergames. The remaining 24 (13 girls; Mean age = 4.88 ± 0.34 years) received conventional physical activity training in the kindergarten as a control group.

Prior to beginning this study, a priori efficacy calculations (effect size f = 0.14; alpha error = 0.05; efficacy = 0.80; correlation between repeated measurements r = 0.80) were done using G*Power 3.1 [35] and revealed a minimal sample size of 36.

### 2.2. Measures

The Early Years Toolbox (EYT) was utilized in this study to assess changes in the EFs of preschool children. The EYT includes a collection of iPad-based measuring activities that may be used to assess EFs in children aged 3 to 5. Preschool children prefer tablet-based tests over traditional paper and pencil assessments [36]. The EYT measuring tasks’ validity and reliability have been established in previous study populations [37,38,39,40]. Specifically, the EYT’s Go/No-Go task has previously exhibited good reliability on both the Go (Cronbach’s α = 0.95) and No-Go (Cronbach’s α = 0.84) trials. Moreover, tasks such as Go/No-Go, Card Sorting, and Mr. Ant had moderately strong correlations (r: 0.40 to 0.80) with existing measures that have been widely used with children (e.g., the National Institute of Health Toolbox, British Ability Scales, etc.), indicating good convergent validity of the EYT with other existing measures [40].

For the current study, three of these tasks were chosen to test inhibition (“Go/No-Go”), shifting (“Card Sorting”), and visual-spatial working memory (“Mr. Ant”). This choice was taken to guarantee that the overall measuring time for each child did not exceed 20 min, with each test requiring around 5 min to complete and higher scores on each task indicating greater EFs performance.

#### 2.2.1. Go/No-Go

The inhibition task requires participants to click on the screen when a fish appears (“Go” trial) and to refrain from clicking when a shark emerges (“Avoid capturing a shark” trial, i.e., “No-Go” trial). The majority of trials (80%) were ‘Go’ trials, which resulted in a predefined proclivity for individuals to respond that had to be inhibited in the ‘No-Go’ trials (20%). The metric for inhibition control was calculated as the product of the Go accuracy and the No-Go accuracy (% Go Accuracy × % No-Go Accuracy).

#### 2.2.2. Card Sorting

Participants in the Shifting challenge had to sort cards (red bunnies or blue boats) into two castles with different logos (blue bunnies or red boats) according to one sorting rule (color or shape), then switch to the other rule. The participant’s ability to correctly transition between classification rules was reflected in the final score, which was the number of right classifications after the first stage.

#### 2.2.3. Mr. Ant

Participants in the WM task were asked to remember the spatial location of a ‘sticker’ that appeared on a cartoon ant, which was presented for five seconds, followed by a four-second blank screen, after which participants were asked to recall and click on the spatial location of the previous sticker on an ant without the ‘sticker’. The task’s difficulty is determined by the number of ‘stickers’ presented (from one to eight stickers), and there were three attempts at the same degree of difficulty, with failure on all three attempts resulting in the termination of the task. The WM capacity indicator was computed by adding 1 point for at least two accurate attempts out of three for each level, beginning with the first, and 1/3 point for just one correct attempt.

#### 2.2.4. Children’s Acceptance of the Exergames

**Figure 1:**
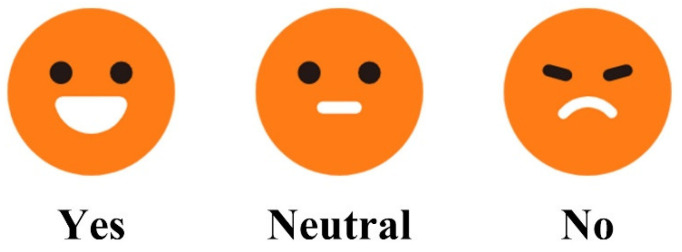
Three-point ordinal Smileyometer scale.

After the intervention, the children in the exergames group were interviewed to acquire a better understanding of their acceptance of and feedback on exergames. Duh et al. [41] developed the interview questions based on the Technological Acceptance Model (TAM) [42] and studied six dimensions: children’s perceived ease of use, perceived usefulness, attitudes toward the game, intention to continue playing the game, technological anxiety about the game, and satisfaction with the game. Three-point ordinal scales were used for the questions; 1-’yes’, 2-’neutral’, and 3-’no’. A Smileyometer was utilized to help the preschool children distinguish between the different options by using different faces to refer to the options (Figure 1). Previous research with children has established the Smileyometer’s validity [41,43].

### 2.3. Procedure

A randomized controlled trial (RCT) was employed in this study to measure the effectiveness of a combined physical–cognitive intervention training using exergames by randomly assigning participants to either the intervention or the control group. Randomized controlled trials are characterized by the fact that by randomly assigning participants, known or unknown differences between participants (e.g., differing levels of physical activity during leisure time between participants) can be statistically controlled. This study used a randomization procedure for grouping and a blinded control to avoid bias.

Specifically, participants were recruited from multiple classes at the same grade level and were grouped using a simple randomization procedure. During the intervention, physical activity training for the exergames and control groups was carried out by separate groups of teachers who had been trained and guided by the researcher. All measuring procedures were performed by an independent measurement team that only visited the kindergarten during measurement and was not ordinarily involved in the intervention training process. In this way, it was ensured that all children, instructors, and researchers were blinded to the greatest extent feasible.

**Figure 2:**
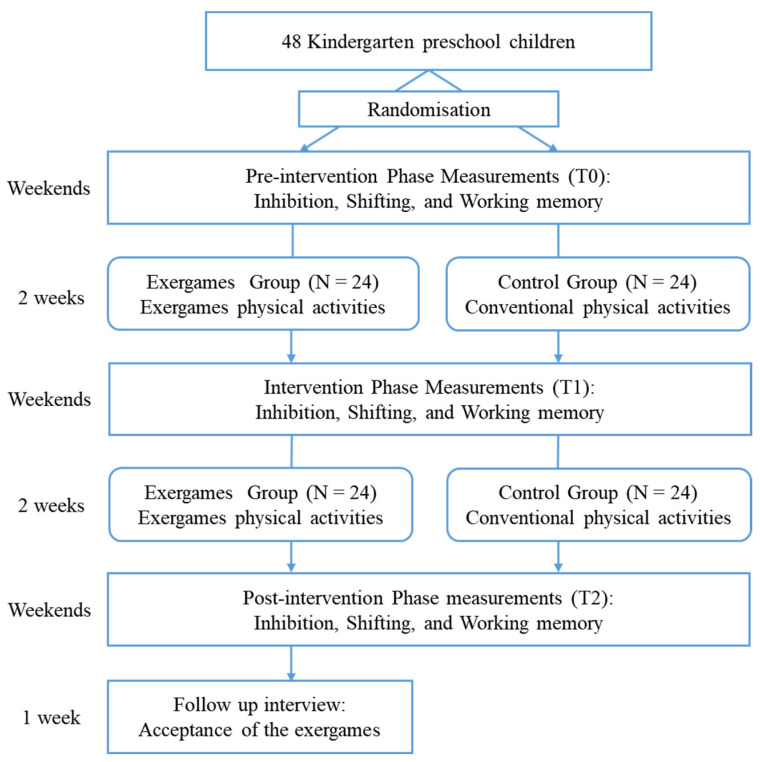
Experimental flow chart of the study.

All participants went through a four-week physical activity training program that included the combined physical–cognitive intervention training for the exergames group and conventional physical activity training for the control group at the same time, with both activities being of equal length. In this study, the children’s EFs (inhibition, shifting, and working memory) were tested at three time points: pre-intervention baseline (T0), after two weeks of intervention (T1), and after the four-week intervention was completed (T2). All three measures were conducted on Saturday and Sunday afternoons to avoid disturbing the children’s normal Monday–Friday sessions and intervention sessions. Additionally, children in the exergames group were also interviewed one week following the intervention period to evaluate their acceptance of the exergames (see Figure 2).

The above experimental procedure was approved by the Ethical Committee of Wenzhou University (WZU-2022-052) and is in accordance with the Declaration of Helsinki.

### 2.4. Intervention

#### 2.4.1. Exergames Physical Activities Condition

The study team collaborated with kindergarten administrators and instructors to include exergames in the intervention group’s preschool physical education curriculum. Specifically, the study team installed the exergame system in four vacant kindergarten classrooms; it included a Nintendo Switch console, four versions of the Just Dance [44] game software (2018, 2019, 2020, and 2021), a 55-inch monitor (resolution 3840 × 2160), and all necessary auxiliary supplies. The Just Dance series was chosen because it has an exclusive “Kids Mode” with eight dance tunes created just for children [45]. This model has been validated in previous research [45,46,47,48], which has shown that it can be practiced by preschool children and is quite popular with them.

The exergames group of 24 children was divided into four classrooms, each accommodating six children holding handles for exergames physical activity training. Each classroom was monitored and organized by a trained instructor. The period was 30 min, Monday through Friday, from 9:30 a.m. to 10:00 a.m., with 5 to 10 min for preparation and warm-up/relaxation. Each week a new version of the game was used to practice a new dance, for a total of 20 lessons spread over four weeks.

#### 2.4.2. Conventional Physical Activities Condition

The conventional physical activity program (e.g., tag games, football, chasing) that was previously created and implemented in the kindergarten was employed by the control group. Sessions were monitored and organized by the classroom instructor and occurred at the same time as the exergames group, either indoors or outside depending on the weather.

## 3. Result

### 3.1. Data Screening and Analysis Plan

All 48 participants completed the EFs test at three time points: the pre-intervention phase (T0), intervention phase (T1), and post-intervention phase (T2); therefore 144 raw data were collected for each of the three EFs assessments. Of the follow-up acceptance interviews, one participant withdrew mid-interview, and one participant took a leave of absence from the interviews, resulting in a total of 22 acceptance interview data collected.

The raw data from the EYT Go/No-Go task was screened first to ensure that only valid responses were included in the subsequent analysis stage. The exclusion criteria [40] were as follows: removal of trials with too rapid a response (response times < 300 ms are unlikely to be a response to the stimulus); removal of non-responsive blocks (blocks with GO accuracy < 20% and NO-GO accuracy > 80%); and removal of blocks with indiscriminate responses (blocks with GO accuracy > 80% and NO-GO accuracy < 20%). The screening did not result in a complete loss of data for any participant, but six participants (<5% of the total) had one of the three Go/No-Go data blocks removed as a result. In this case, the inhibitory control index for that participant was calculated using the remaining two blocks [39].

The next major analysis constructed linear mixed-effects models for each of the EFs measurement variables to examine the effect of intervention training on EFs. The models included fixed effects for the group (exergames and control), time (T0, T1, and T2), and the interaction between group and time; participant intercepts were included as random effects [49] and were age controlled. The variance components were estimated using restricted maximum likelihood (REML) methods, and the degrees of freedom were calculated using the Satterthwaite adjustment. The model data were reported using the best practice reporting style recommended by Meteyard and Davies [50], with the goodness of fit of the linear mixed-effects model given as Marginal and Conditional R^2^ [51]. Before analysis, Shapiro-Wilk tests and visual inspection of Q-Q plots suggested that the standardized residual distributions of all outcome variables had approximately normal distributions. The Least Significant Difference (LSD) was used as a post hoc test, and differences were judged to be statistically significant when the probability was less than 5% (*p* < 0.05). Jamovi 2.3 was used to conduct the statistical analyses [52,53].

### 3.2. Analysis of Intervention Effects

**Figure 3:**
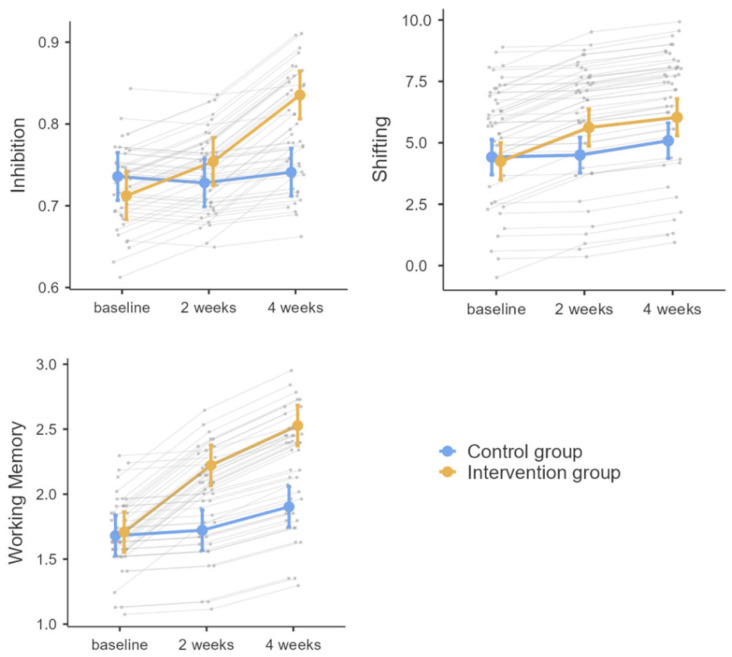
The executive function test score means over time and the random effects of participant intercepts.

Table 1 shows descriptive statistics for the EFs measurements results. Figure 3 depicts the trend in the estimated means of the EFs measurement scores over time for the two groups.

#### 3.2.1. Inhibition

For Inhibition, the results of the linear mixed-effects model were estimated as shown in Table 2. At baseline, there was no statistically significant difference in measurement scores between the exergames and control groups (*p* = 0.564). After 2 weeks of intervention training, the group-by-time interaction revealed no statistically significant difference in the magnitude of change in measurement scores (from T0 to T1) between the two groups (*p* = 0.345). However, after 4 weeks of intervention training, the exergames group’s increase in scores (from T0 to T2) was on average 0.118 larger than the control group’s, and there was a statistically significant difference in training gains between the two groups (β = 0.118, *p* = 0.026). As shown in Table 3, the post-hoc analysis revealed that after four weeks of intervention training, the exergames group had a significant increase in measured scores compared to baseline levels (*p* = 0.001), but the control group had no significant difference in pre- and post-scores (*p* = 0.887). For the linear mixed-effects model, fixed effects explained 7% of the variation in Inhibition (R^2^ Marginal = 0.0721), whereas the entire model (fixed plus random effects) explained 28% of the variance (R^2^ Conditional = 0.2795).

In general, the combined physical–cognitive intervention training using exergames significantly improved the participants’ performance on the Inhibition task. As shown in Figure 3, inhibition scores in the exergames group exhibited a continuously increasing trend from T0 to T2, with an accelerating trend from T1 to T2, but inhibition scores in the control group showed a consistently flat trend.

#### 3.2.2. Shifting

For Shifting, the results of the linear mixed-effects model were estimated as shown in Table 4. At baseline, there was no statistically significant difference in measurement scores between the exergames and control groups (*p* = 0.830). The group by time interaction revealed that following 2 weeks of intervention training, the exergames group’s scores (from T0 to T1) increased on average 1.292 points more than the control group’s, with a statistically significant difference in training gains between the two groups (β = 1.292, *p* = 0.011). After four weeks of intervention training, the exergames group maintained this advantage (β = 1.125, *p* = 0.027). As shown in Table 3, the post-hoc analysis revealed that after the first two weeks of intervention training, the exergames group had a significant increase in scores compared to the baseline (T0 to T1: *p* < 0.001), whereas the second two weeks of intervention training had no significant influence on scores (T1 to T2: *p* = 0.242). The control group, on the other hand, had no significant differences in scores before and after training (T0 to T1: *p* = 0.814; T0 to T2: *p* = 0.063). For the linear mixed-effects model, fixed effects explained 10% of the variation in Inhibition (R^2^ Marginal = 0.105), whereas the entire model (fixed plus random effects) explained 82% of the variance (R^2^ Conditional = 0.822).

In general, the combined physical–cognitive intervention training using exergames significantly improved the participants’ performance on the Shifting task. As shown in Figure 3, Shifting scores in the exergames group exhibited a continuously increasing trend from T0 to T2, with a slowing trend from T1 to T2, but Shifting scores in the control group showed a consistently flat trend.

#### 3.2.3. Working Memory

For Working Memory, the results of the linear mixed-effects model were estimated as shown in Table 5. At baseline, there was no statistically significant difference in measurement scores between the exergames and control groups (*p* = 0.947). The group-by-time interaction showed that after 2 weeks of intervention training, there was no significant difference in the magnitude of change in measurement scores (from T0 to T1) between the two groups (*p* = 0.085). However, after 4 weeks of intervention training, the exergames group’s increase in scores (from T0 to T2) was on average 0.118 larger than the control group’s, and there was a statistically significant difference in training gains between the two groups (β = 0.597, *p* = 0.030). As shown in Table 3, the post-hoc analysis revealed that after the first two weeks of intervention training, the exergames group had a significant increase in scores compared to the baseline (T0 to T1: *p* = 0.009), whereas the second two weeks of intervention training had no significant influence on scores (T1 to T2: *p* = 0.114). The control group, on the other hand, had no significant differences in scores before and after training (T0 to T1: *p* = 0.828; T0 to T2: *p* = 0.249). For the linear mixed-effects model, fixed effects explained 16% of the variation in Inhibition (R^2^ Marginal = 0.157), whereas the entire model (fixed plus random effects) explained 36% of the variance (R^2^ Conditional = 0.364).

In general, the combined physical–cognitive intervention training using exergames significantly improved the participants’ performance on the Working Memory task. As shown in Figure 3, Working Memory scores in the exergames group exhibited a continuously increasing trend from T0 to T2, with a slowing trend from T1 to T2, but Working Memory scores in the control group showed a consistently flat trend.

### 3.3. Children’s Acceptance of the Exergames

Table 6 shows the results for the preschool children’s acceptance of exergames. The results indicate that the exergames were well-accepted, with over 80% of children finding them simple to use, and they intended to continue using them in future physical activities. Almost all children expressed enjoyment and did not find the game to be boring.

## 4. Discussion

This study examined the effects of combined physical–cognitive intervention training using exergames on preschool children’s EFs in a randomized controlled trial. The exergames physical activities significantly enhanced children’s performance on three EFs tasks (inhibition, shifting, and working memory) after four weeks of physical activity training. The exergames training resulted in significantly larger improvements in EFs than conventional physical activities.

This result provides good support for the hypothesis in this study and demonstrates the effectiveness of implementing exergames physical activity training for preschool children. As mentioned in the review of the literature, previous studies in groups such as school-aged children [14,54,55], children with developmental disabilities [29], and older adults [27] have confirmed that combined interventions of physical activities and cognitive challenges are more effective than purely aerobic exercise interventions in terms of enhancing EFs. The present study further replicates this finding for preschool children aged 4–5 years old enrolled in kindergarten, thereby adding empirical evidence for this area of research.

An interesting finding in this study is that EFs were not significantly increased in the conventional physical activities group over the intervention period, which contradicts earlier research concluding that longitudinal physical activity programs improved EFs [56,57]. This finding may be related to the short intervention period of only four weeks in this study; conventional physical activities with less cognitive involvement would barely have a significant influence on EFs in such a short period. Surprisingly, the exergames intervention program, which demands a great deal of cognitive engagement, showed a significant improvement in EFs even in a shorter intervention time. In the test during the intervention phase (T1) after just two weeks of intervention, EFs in the exergames group showed a significant tendency towards improvement. Several factors may explain this observation. Firstly, preschool children are in a critical period of rapid EFs development and brain development [10,32,33], so combined physical–cognitive interventions that require significant attention and cognitive effort from children are more effective ways of improving EFs than in adults, older adults, and others. Secondly, exergames such as games provide children with a positive emotional experience and enjoyment, thus increasing their intrinsic motivation to exercise. Compared to conventional sports, children who use exergames are more motivated to exercise and more engaged during exercise [27,58]. The results of post-intervention interviews also revealed that almost all children enjoyed the exergames utilized in this study and desired to continue using them for physical activities.

## 5. Limitations and Future Research

Some limitations mean that the results of this study should be interpreted with caution. The fact that the participants in this study came from the same kindergarten and were comprised of children of relatively well-educated parents, and that the study had a relatively small sample size may have affected the generalizability of the results and thus prevented full extension to a larger sample or other groups. Furthermore, the study had a short intervention period, making it impossible to rule out the potential that the exergame group’s improved performance was impacted by the novelty of the exergames. Although exergames showed a significant advantage over conventional physical activities at this time, this result may change if the intervention period was extended.

It is thus advised that further research on the present issue be conducted over a longer period with a larger sample group to confirm and validate the current findings and to assess the long-term effects of exergames on children. Furthermore, it would be exciting to validate the findings outside of the preschool setting. Incorporating exergame into the home setting for parent-child play, for instance, might promote communication and bonding between parents and children and have a positive effect on the cognition and learning of children. Finally, future research in the field of early childhood might include mixed types of studies, which would improve the interpretability of the findings.

## 6. Conclusions

Following a four-week intervention training period, 4–5-year-old preschool children who received the exergames physical activity training program showed significantly greater gains in all three EFs tasks (inhibition, shifting, and WM) when compared to the conventional physical activities program, as well as good acceptance of exergames.

The results of this study show the feasibility and effectiveness of incorporating exergames to improve preschool children’s EFs in kindergarten, supporting previous findings and providing additional empirical evidence from early childhood research. Further research in this area is necessary to explore the long-term effectiveness and safety of exergames.

## Figures and Tables

**Table 1:** Descriptive statistics of executive function test scores at each time point for the control and exergames groups.

Executive Functions	Time	Control Group (*n* = 24)		Exergames Group (*n* = 24)	
		Mean	*SD*	Mean	*SD*
Inhibition (Go/No-Go)	T0	0.74	0.14	0.71	0.16
	T1	0.73	0.15	0.75	0.16
	T2	0.74	0.14	0.84	0.11
Shifting (Card Sorting)	T0	5.21	3.15	5.13	2.94
	T1	5.29	3.43	6.50	2.45
	T2	5.88	2.40	6.92	2.21
Working Memory (Mr. Ant)	T0	1.68	0.76	1.71	0.77
	T1	1.72	1.02	2.22	0.46
	T2	1.90	0.76	2.53	0.73

SD, standard deviation; T0, pre-intervention phase; T1, intervention phase; T2, post-intervention phase.

**Table 2:** Summary of the Linear mixed model for the Inhibition scores.

	Estimate	SE	95% CI	*t*	*p*
**Fixed Effects**					
(Intercept)	0.736	0.029	(0.678, 0.794)	24.95	<0.001
Age	0.018	0.048	(−0.075, 0.111)	0.38	0.704
Group Effect ^a^	−0.024	0.042	(−0.106, 0.058)	−0.58	0.564
Group * Time Effect ^b^ (T1)	0.049	0.053	(−0.053, 0.151)	0.95	0.345
Group * Time Effect ^b^ (T2)	0.118	0.053	(0.016, 0.220)	2.27	0.026
			**Variance**	**SD**	**ICC**
**Random Effects**					
Subject (Intercept)			0.0047	0.068	0.224
Residual			0.0162	0.127	

R^2^ Marginal: 0.0721; R^2^ Conditional: 0.2795; SE, standard error; CI, confidence interval. Model equation: Inhibition ~ 1 + age + time + group + time:group + (1|subject). ^a^ Group effect is defined as the between-group difference between the experimental and control groups at baseline. ^b^ Group * Time effect is defined as the group difference between the experimental and control groups in the magnitude of change in scores at T1 and T2 time points relative to the baseline at T0.

**Table 3:** Post-hoc comparison of executive function measurements at different time points.

Comparison	Groups (*n* = 24)	Difference	SE	*t*	*p*
**Inhibition**					
T0 → T1	Intervention group	0.042	0.037	1.13	0.26
	Control group	−0.008	0.037	−0.21	0.835
T1 → T2	Intervention group	0.081	0.037	2.22	0.029
	Control group	0.013	0.037	0.35	0.726
T0 → T2	Intervention group	0.123	0.037	3.35	0.001
	Control group	0.005	0.037	0.14	0.887
**Shifting**					
T0 → T1	Intervention group	1.375	0.354	3.89	<0.001
	Control group	0.083	0.354	0.24	0.814
T1 → T2	Intervention group	0.417	0.354	1.18	0.242
	Control group	0.583	0.354	1.65	0.103
T0 → T2	Intervention group	1.792	0.354	5.07	<0.001
	Control group	0.667	0.354	1.89	0.063
**Working Memory**					
T0 → T1	Intervention group	0.514	0.192	2.68	0.009
	Control group	0.042	0.192	0.22	0.828
T1 → T2	Intervention group	0.306	0.192	1.60	0.114
	Control group	0.181	0.192	0.94	0.348
T0 → T2	Intervention group	0.819	0.192	4.28	<0.001
	Control group	0.222	0.192	1.16	0.249

SE, standard error; T0, pre-intervention phase; T1, intervention phase; T2, post-intervention phase.

**Table 4:** Summary of the Linear mixed model for the Shifting scores.

	Estimate	SE	95% CI	*t*	*p*
**Fixed Effects**					
(Intercept)	5.252	0.561	(4.153, 6.352)	9.36	<0.001
Age	2.109	1.211	(−0.264, 4.482)	1.74	0.088
Group Effect	−0.171	0.794	(−1.728, 1.385)	−0.22	0.830
Group * Time Effect (T1)	1.292	0.50	(0.311, 2.27)	2.58	0.011
Group * Time Effect (T2)	1.125	0.50	(0.145, 2.11)	2.25	0.027
			**Variance**	**SD**	**ICC**
**Random Effects**					
Subject (Intercept)			6.04	2.46	0.801
Residual			1.50	1.23	

R^2^ Marginal: 0.105; R^2^ Conditional: 0.822; SE, standard error; CI, confidence interval. Model equation: Shifting ~ 1 + age + time + group + time:group + (1|subject).

**Table 5:** Summary of the Linear mixed model for the Working Memory scores.

	Estimate	SE	95% CI	*t*	*p*
**Fixed Effects**					
(Intercept)	1.687	0.156	(1.381, 1.993)	10.81	<0.001
Age	0.312	0.255	(−0.189, 0.812)	1.22	0.229
Group Effect	0.015	0.221	(−0.418, 0.448)	0.067	0.947
Group * Time Effect (T1)	0.472	0.271	(−0.059, 1.003)	1.74	0.085
Group * Time Effect (T2)	0.597	0.271	(0.066, 1.128)	2.21	0.030
			**Variance**	**SD**	**ICC**
**Random Effects**					
Subject (Intercept)			0.144	0.379	0.246
Residual			0.440	0.664	

R^2^ Marginal: 0.157; R^2^ Conditional: 0.364; SE, standard error; CI, confidence interval. Model equation: Working Memory~1 + age + time + group + time:group + (1|subject).

**Table 6:** Results of a survey on young children’s acceptance of the exergames.

	Questions	Yes	Neutral	No
Perceived usefulness	Do you think that playing this game is useful to you?	11 (50%)	6 (27%)	5 (22%)
Perceived ease of use	Do you think this game is easy to play?	18 (82%)	1 (5%)	3 (14%)
Attitude	Would you like to play this game in your kindergarten?	21 (95%)	0 (0%)	1 (5%)
Intention	Would you like to continue using this game to learn more dances?	18 (82%)	3 (14%)	1 (5%)
Anxiety	Do you think this dance game is boring?	2 (9%)	1 (5%)	19 (86%)
Satisfaction	Is this dance game satisfying to you?	19 (86%)	2 (9%)	1 (5%)

## Data Availability

The data presented in this study are available on request from the corresponding author.
